# *Pasteurella multocida* Toxin Activates Various Heterotrimeric G Proteins by Deamidation

**DOI:** 10.3390/toxins2020205

**Published:** 2010-01-28

**Authors:** Joachim H. C. Orth, Klaus Aktories

**Affiliations:** Institute for Experimental and Clinical Pharmacology and Toxicology, University of Freiburg, 79104 Freiburg, Germany; Email: Joachim.Orth@pharmakol.uni-freiburg.de

**Keywords:** G protein, α-subunit, deamidation, GTPase, Gα_q_, Gα_i_, Gα_12/13_

## Abstract

*Pasteurella multocida* produces a 146-kDa protein toxin (*Pasteurella multocida* toxin, PMT), which stimulates diverse cellular signal transduction pathways by activating heterotrimeric G proteins. PMT deamidates a conserved glutamine residue of the α-subunit of heterotrimeric G proteins that is essential for GTP-hydrolysis, thereby arresting the G protein in the active state. The toxin substrates are Gα_q_ Gα_13_ and the Gα_i_-family proteins. Activation of these α-subunits causes stimulation of phospholipase Cβ, Rho-guanine nucleotide exchange factors or inhibition of adenylyl cyclase. This article provides the current knowledge on PMT concerning the structure-function analysis based on the crystal structure and recently elucidated molecular mode of action. Furthermore, the impact of PMT on cellular signaling is discussed.

## 1. Introduction

*Pasteurella multocida* is a Gram negative opportunistic pathogenic bacterium living in the nasal, pharyngeal space of animals. Human infections occur usually by scratches and bites of domesticated animals (e.g., mainly cats and dogs). In addition, contact with salvia is sufficient for colonization of bacteria [1]. *P. multocida* is of particular importance in livestock management, especially of pigs. The infection of swine with *P. multocida* leads under special conditions to a atrophic rhinitis that was first described by Franque in 1830 [2]. A main symptom in pigs is the loss of nasal turbinate bones leading to a twisted or shortened snout. The causative agent of atrophic rhinitis is a protein toxin produced by *P. multocida* (*Pasteurella multocida* toxin, PMT) [3,4].

## 2. Signal Transduction and Molecular Mechanism of PMT

**Figure 1:**
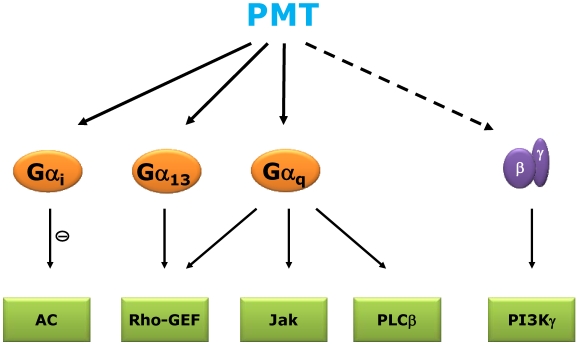
Scheme of PMT-activated heterotrimeric G proteins and subsequent signaling pathways. PMT activates Gα_i_ to inhibit adenylyl cyclase (AC). PMT-induced activation of Gα_13_ and Gα_q_ stimulates Rho guanine nucleotide exchange factors (RhoGEF) leading to the activation of the low molecular mass GTPase RhoA. Moreover, activation of Gα_q_ leads to stimulation of the Jak-STAT (Janus kinase, signal transducer and activator of transcription) pathway and the canonical Gα_q_ effector phospholipase Cβ (PLCβ). By activation of the Gα-subunit, Gβγ is released from the heterotrimeric complex and mediates signaling via βγ-specific effectors, e.g., phosphoinositide-3-kinase (PI3K)γ.

PMT stimulates a variety of signal transduction pathways (Figure 1). Phospholipase C (PLC) β, which is activated by Gα_q_, is stimulated to catalyze inositol trisphosphate production and Ca^2+^ mobilization. In addition, the small GTPase RhoA is activated leading to stress fiber formation. Moreover, PMT is a strong mitogen in various cell lines. These effects are mainly due to the activation of heterotrimeric G proteins of the Gα_q_ and Gα_12/13_ family. Furthermore, PMT activates Gα_i_ thereby inhibiting adenylyl cyclase. Toxin-induced activation of heterotrimeric G proteins (e.g., G_i_) releases Gβγ, which stimulates signaling pathways.

PMT was found to be an extremely potent mitogen for several cell types. Concentrations as low as 1 to 2 pM stimulate DNA synthesis [5]. However, the toxin induces proliferation of osteoblasts even though one of the PMT-induced symptoms is the atrophy of nasal turbinate bones. The underlying signaling pathways that govern PMT-induced proliferation are largely unknown. In HEK293 cells, PMT transactivates the epidermal growth factor (EGF) receptor in a Gα_q_-dependent manner. However, in cardiomyocytes the stimulation of the MAP kinase pathway occurs in an EGF receptor-independent manner [6,7]. Therefore, the PMT-induced pathways to enhance proliferation seem to be cell type specific.

PMT enhances inositol trisphosphate production by activation of PLCβ, which in turn is stimulated by α-subunits of the Gα_q/11_ family [5,8]. Studies with Gα_q_ and Gα_11_ gene deleted mouse embryonic fibroblasts (MEF) showed that PMT activates PLCβ only via Gα_q_ but not via the closely related Gα_11_[9]. This observation is noteworthy given that Gα_q_ and Gα_11_ are 89% identical at the amino acid sequence level and Gα_q_-coupled receptors are generally found to activate Gα_11_ too. PMT distinguishes between both G proteins on the basis of the helical domain, which is inserted into the highly conserved GTPase domain. Replacement of helix αB of Gα_11_ by the Gα_q_ helix enabled the toxin to activate the mutant Gα_11_ protein. However, introduction of helix αB of Gα_11_ into Gα_q_ leads to a chimera, which is not activated by PMT [10].

Besides the stimulation of the prototypical Gα_q_ effector PLCβ, the toxin stimulates the Jak-STAT (Janus kinase, signal transducer and activator of transcription) pathway in a Gα_q_-dependent manner [11]. PMT activates STAT1, 3 and 5 leading to altered gene expression, e.g. up-regulation of cancer-associated cyclooxygenase (COX)-2 expression.

RhoA is indirectly activated by PMT. The PMT-induced activation of the small GTPase RhoA is functionally connected with different families of heterotrimeric G proteins. It is known that the Gα_12/13_ and Gα_q_ families are capable of activating RhoA via guanine-nucleotide exchange factors (GEFs) like p115RhoGEF and p63RhoGEF, respectively [12,13]. Studies with Gα_q/11_-deficient MEF showed that PMT stimulates RhoA in a Gα_q_-independent manner [9]. Overexpression of Gα_13_ in Gα_12/13_-deficient MEF together with pharmacological inhibition of Gα_q_ restored PMT-induced stimulation of RhoA [14]. However, comprehensive studies with Gα_q/11_- and Gα_12/13_-deficient MEF revealed that the toxin utilizes both families, Gα_12/13_ and Gα_q_, for stimulation of the small G protein RhoA.

Beside Gα_q_ and Gα_12/13_ the toxin activates Gα_i_. This leads to inhibition of the adenylyl cyclase [15]. PMT not only activates the α-subunit of heterotrimeric G proteins, but also stimulates Gβγ-pathways by release of Gβγ from G proteins. For example, PMT causes activation of phosphoinositide-3-kinase (PI3K)γ, which is a prototypical effector of Gβγ [16].

The recognition of Gα_i_ as a target of PMT was one of the experimental milestones for elucidation of the molecular mechanism of PMT. Gα_i_ is much easier to study than Gα_q_ or Gα_12/13_, because recombinant expression in *E. coli* is possible. Furthermore, its GTPase function (e.g., GTP hydrolyzing activity) can be measured easily.

**Figure 2:**
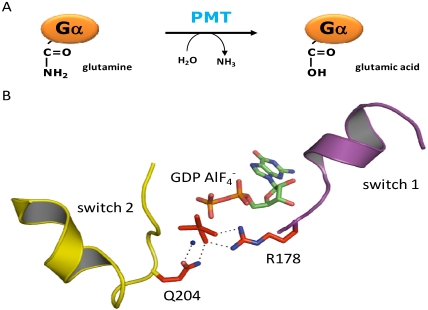
(a) PMT deamidates the essential glutamine residue of the α-subunit of heterotrimeric G proteins, resulting in a glutamic acid residue. (b) Overview of Gα_i1_ complexed with GDP/AlF_4_^-^ mimicking the transition state of the hydrolysis of the γ-phosphate of GTP. Two essential amino acid residues are highlighted: Arginine-178 in switch 1 (magenta) and glutamine-204 (corresponding to glutamine-205 in Gα_i2_) in switch 2 (yellow). Both residues are involved in coordinating the γ-phosphate (here AlF_4_^-^) and a water nucleophile (blue sphere). PMT deamidates the glutamine residue in switch 2. Therefore, hydrolysis of the γ-phosphate of GTP is not possible anymore and the G protein is arrested in the active state. This image was generated using PyMol and PDB data file 1GFI.

To analyze the molecular mechanism of PMT, the toxin and Gα_i2_ were recombinantly co-expressed in *E. coli*. Studying Gα_i2_ from co-expression with active toxin showed inhibition of basal and RGS (regulator of G protein signaling)-stimulated GTPase activity. By tandem-mass spectrometry a difference in mass of one Dalton at glutamine-205 was detected, indicating a deamidation (Figure 2), which results in a glutamic acid residue [17]. This glutamine residue is conserved throughout the GTPase superfamily and is essential for the hydrolysis of the γ-phosphate of GTP thereby terminating the active state of the GTPase [18]. Deamidation of the pivotal glutamine residue results in an inhibition of the GTPase activity and arrests the G protein in a permanent active state. Functional equivalent to glutamine-205 of Gα_i2_ is glutamine-209 of Gα_q_ and Gα_11_. The deamidation of Gα_q_, which results in a change of the isoelectric point, could be demonstrated by faster migration in native gel electrophoresis. In agreement to the resistance of Gα_11_ towards PMT, no toxin-catalyzed deamidation of Gα_11_ was detectable [17].

PMT activates heterotrimeric G proteins independent of any receptor interaction. A chimera of a GPCR (α_1_-adrenoceptor) with Gα_q_ as well as a deletion mutant of Gα_q_, incapable of interacting with GPCRs is stimulated by PMT [19]. Accordingly, ADP-ribosylation of Gα_i_ by pertussis toxin, which uncouples the G protein from GPCRs, has no impact on subsequent PMT-induced activation. On the other hand pre-treatment of G_i_ with PMT inhibits pertussis toxin-induced ADP-ribosylation [15]. This is explained by the fact that pertussis toxin modifies the inactive, heterotrimeric G_i_ complex [20]. Therefore, activation and dissociation of the heterotrimeric G protein by PMT leads to insensitivity towards pertussis toxin.

## 3. Structure

PMT is a 146-kDa protein toxin, encompassing 1,285 amino acid residues [21]. It belongs to a family of dermonecrotic toxins, including the cytotoxic necrotizing factors (CNF1-3) from *Escherichia coli* and *Yersinia pseudotuberculosis* (CNFy) and dermonecrotic toxin (DNT) from *Bordetella pertussis* and *B. bronchiseptica* and PMT. These toxins share significant sequence homology in their N-terminal parts. In CNFs and DNT, the N-terminus is important for receptor binding and translocation to the cytosol [22,23]. Therefore, the homology between PMT and CNFs / DNT suggests a similar function of the N-terminal part and overall a similar domain structure with a receptor binding and translocation domain in the N-terminus and a catalytic domain in the C-terminus. 

**Figure 3:**
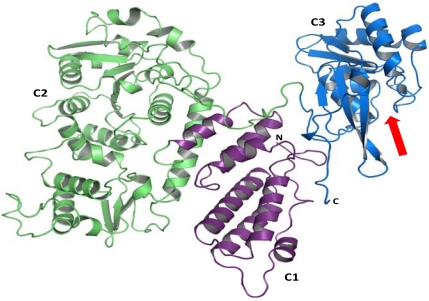
Domain architecture of the C-terminal part of PMT. The crystal structure of the C-terminal part of PMT (amino acids 575-1285) revealed 3 domains C1, C2 and C3. The C1 domain (magenta) has similarities with a domain of *Clostridium difficile* toxin B and targets the toxin to the inner part of the plasma membrane. The function of the C2 domain (green) is unknown. The C3 domain provides a catalytic cleft (arrow) and harbors the biological activity of the toxin. This image was generated using PyMol and PDB data file 2EBF.

In line with this model, fragments of PMT that lack the receptor or proposed translocation domains have no toxic effects, when applied to culture medium or admistered to animals [24,25]. However, a C-terminal fragment without receptor binding and translocation domain, consisting of amino acid 581 to 1285, has the identical effects as full-length toxin if directly introduced into mammalian cells by electroporation [23,26]. Recently, this part was crystallized (Figure 3) [27]. The C-terminus of PMT consists of three domains C1, C2 and C3 with a so-called Trojan horse-like overall structure. The C1 domain (residues 569-719) has structural similarities with another bacterial protein toxin, *Clostridium diffcile* toxin B. In toxin B this domain targets the toxin to the intracellular site of the plasma membrane [28]. An identical function is suggested for the PMT C1 domain [27]. The C2 domain (residues 720-1104) is similar to a folylpolyglutamate synthetase but its function remains unknown. Most important is the C3 domain (residues 1105-1285) harboring the catalytic activity of the toxin.

**Figure 4:**
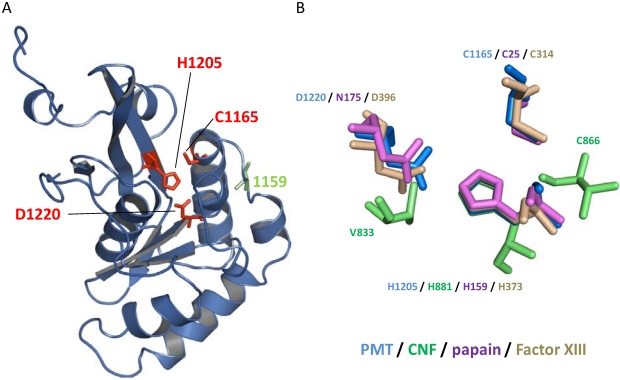
(a) Structure of the C-terminal C3 domain of PMT. This domain harbors the biological activity of the toxin with the catalytic triad (highlighted in red) cysteine-1165, histidine-1205 and aspartic acid-1220. Interestingly, cysteine-1165 of the triad is normally closed by a disulfide bridge with cysteine at position 1159 (green). Here the structure of a mutant toxin (C1159S), which releases the catalytic active cysteine-1165 from the disulfide bond, is shown. (b) Catalytic triads of thiol proteases (papain), transglutaminases (Factor XIII) and deamidases (PMT, CNF1). Catalytic active histidines were superimposed. This image was generated using PyMol and PDB data file 2EBF (PMT), 1HQ0 (CNF1), 1POP (papain) and 1GGT (Factor XIII).

The primary amino acid sequence of the C3 domain showed no homology to any other protein but the crystal structure revealed first insights into the possible function of PMT. Crystallization of wt-PMT leads to a structure harboring a disulfide bond between cysteine-1159 and cysteine-1165. Cleavage of this disulfide bond by mutation of cysteine-1159 to serine leads to reorientation of cysteine-1165 (Figure 3 and Figure 4). The re-orientated cysteine-1165, together with histidine-1205 and aspartic acid-1220, forms a topology that resembles a catalytic triad, suggesting an essential role for these residues in the activity of PMT [27]. Localization of the biological activity in the very *C*-terminus was supported by the finding that the ectopical expression of the C3 domain in mammalian cells leads to identical effects as the expression of the full-length toxin [29].

As already described, PMT acts as a deamidase to constitutively activate heterotrimeric G proteins. The same mechanism is utilized by CNFs, which deamidate the essential glutamine in low molecular mass GTPases as RhoA, Rac or Cdc42 [30,31]. In addition, DNT deamidates Rho proteins at glutamine-61(63) like CNF. Moreover, DNT can act as a transglutaminase on Rho proteins [32,33]. PMT has sequence similarity with CNF and DNT in the N-terminal binding and translocation domains. In contrast, PMT has no obvious structural similarity with CNFs or DNT at the C-terminal catalytic domain [27,34]. Even the topology of the catalytic center is different (Figure 4B). PMT and CNF share the catalytic amino acid residues histidine and cysteine. The third catalytic amino acid residues in the case of PMT is aspartic acid and in the case of CNF valine.

However, the catalytic triads of PMT and papain match perfectly, therefore it was suggested that PMT acts as a cysteine protease. Functional and structural comparison of deamidases and thiol proteases with transglutaminases explains this divergence. From the type of chemical reaction that they catalyze, transglutaminases, which replace the NH_2_‑group of the amide of a glutamine by another amine residue, are closely related to deamidases, which change the amide to a carboxylate by using H_2_0 as co-substrate. Furthermore, thiol proteases like papain catalyze the reverse reaction of transglutaminases. Due to this reason, it is not surprising that the catalytic triad of PMT matches the catalytic triads of thiol proteases or transglutaminases [35,36]. For example, the catalytic triad (Cys-His-Asp) of the transglutaminases factor XIII perfectly matches the position of the catalytic triad of PMT.

## 4. Toxin Uptake

To date, our knowledge about the cellular up-take of PMT is rather limited. It is generally accepted that receptor-mediated endocytosis is essential for PMT uptake by mammalian cells. Uptake could be competed with mixed gangliosides, therefore the receptor was suggested to belong to this class [37]. Release of the toxin into the cytosol from early endosome is driven by acidification; consequently bafilomycin A1, an inhibitor of the vacuolar ATPases, inhibits intoxication [38]. Accordingly, mimicking endosomal acidification by exposing toxin-bound cells to acidic medium leads to direct translocation of PMT to the cytosol. It is suggested that acidification induces conformational changes of PMT allowing exposure of two hydrophobic helices. The actual uptake model suggests that these helices are introduced into the endosomal membrane [39]. The precise mechanism of the translocation of PMT into the cytosol remains to be elucidated.

## 5. Conclusions

For a long time the mechanism of PMT-mediated activation of heterotrimeric G-proteins remained enigmatic. The elucidation of the crystal structure of the toxin and recognition of Gα_i_ as an additional toxin substrate were pivotal to clarify the molecular mechanism of PMT. The toxin deamidates a glutamine of the α-subunits of heterotrimeric G-proteins. This glutamine is essential for hydrolyzing GTP. Therefore, the deamidation leads to a permanent active G protein. PMT acts on different families of heterotrimeric G-proteins (Gα_i_, Gα_q_ and Gα_12/13_), even though it surprisingly distinguishes between the closely related Gα_q_ and Gα_11_. In addition to Gα-signaling, PMT induces signaling by Gβγ subunits, which are released upon activation of heterotrimeric G proteins.

Interestingly, PMT is one of the strongest mitogens known. In addition, it is known that frequent somatic mutations in the gene *Gnaq*, encoding Gα_q_, are found in melanoma of the uvea (46%) and in blue naevi (83%). These mutations result in change of glutamine-209 in a manner similar to the constitutive activation, which is caused by PMT [40]. Therefore, it remains to be clarified whether the toxin plays a role in cancer development.
